# Coordination Patterns and Energy Flow Analysis in Sit-to-Stand Transitions Among Individuals with Different Body Mass Indexes

**DOI:** 10.3390/life15030464

**Published:** 2025-03-14

**Authors:** Lei Li, Xuan Liu, Ye Liu

**Affiliations:** 1 School of Sport Science, Beijing Sport University, Beijing 100084, China; lilei120800@163.com (L.L.); liuxuan_12354@163.com (X.L.); 2 Laboratory of Sports Stress and Adaptation of General Administration of Sport, Beijing Sport University, Beijing 100084, China

**Keywords:** body mass index, sit-to-stand, coordination pattern, energy flow

## Abstract

Background: This study investigates the differences in limb coordination patterns and energy transfer strategies during sit-to-stand (STS) transitions among young adults (18–30 years) with overweight (OW), normal weight (NW), and underweight (UW) conditions, providing a theoretical foundation for understanding the impact of BMI variations on movement control mechanisms and informing health intervention strategies. Methods: Forty participants were classified into OW, NW, and UW groups. Motion data were collected via an infrared motion capture system and force plate. Biomechanical indices were computed using Visual 3D and MATLAB2020a. Coordination patterns were assessed using vector coding, and the segmental net power was analyzed to evaluate energy flow during STS. Statistical analyses were performed using one-way ANOVA (α = 0.05). Results: Compared to the NW and UW groups, the OW group exhibited significant differences in movement coordination patterns and energy flow. In terms of coordination patterns, the OW group adopted more hip-knee distal coordination patterns in the FMP phase and more knee-ankle proximal coordination patterns. In the MTP phase, the OW group exhibited a lower frequency of hip-ankle anti-phase coordination patterns compared to the UW group. In the EP phase, the OW group showed a lower frequency of trunk-pelvis proximal coordination patterns than the UW group (*p* < 0.05). Regarding energy flow, in the FMP phase, the OW group exhibited higher joint power (JP) and segment power (SP) in the trunk compared to the UW group. In the pelvic segment, both JP and SP were higher in the OW group than in the NW and UW groups. In the thigh segment, muscle power (MP) was higher in the OW group than in the NW and UW groups, and SP was higher than in the NW group (*p* < 0.05). Conclusion: Changes in BMI affect movement coordination and energy transfer strategies during STS. OW individuals compensate for insufficient hip drive by relying on trunk and pelvic power, which may increase the knee and trunk load over time. In contrast, UW individuals exhibit greater lower-limb flexibility and rely on trunk-pelvis coordination to compensate for stability deficits. Future research should develop targeted exercise interventions to optimize movement patterns and reduce injury risk across BMI groups.

## 1. Introduction

Body mass index (BMI) is widely recognized as a critical indicator of physical health and a key component of the World Health Organization’s (WHO) fracture risk assessment framework [1]. Based on BMI classifications, individuals are typically categorized into four groups: obese, overweight, normal weight, and underweight. While obesity has been extensively studied due to its associations with osteoarthritis and other metabolic disorders [2], both overweight and underweight conditions introduce specific musculoskeletal adaptations that can significantly alter movement patterns, warranting comparable attention [3,4].

Studies have found that obese individuals exhibit gait instability and decreased postural control during walking. As a precursor to obesity, overweight individuals also demonstrate a forward shift in the center of mass (COM) during movement [5], which increases the load on the lower-limb joints and affects balance and gait stability. Additionally, a low body weight may also lead to changes in gait and postural control during movement [6]. These findings suggest that changes in the body mass index (BMI) can influence human movement control. However, a study by Veronica et al. [3] found no significant differences in the spatiotemporal walking parameters among overweight, normal weight, and underweight adolescents. This finding suggests that isolated joint movement time series may offer limited insight into how underlying segments coordinate to produce limb posture and distribute mechanical loads. By evaluating the coordination patterns of the trunk and lower limbs, a more comprehensive understanding of segmental interactions and their role in movement organization can be achieved [7,8].

Understanding segmental interactions requires not only identifying the states of interaction but also exploring how these states are achieved. From an energy perspective, segmental interactions are facilitated through the coordinated application of active forces (neuromuscular actions) and passive forces (inter-joint dynamics), enabling efficient energy transfer between body segments [9,10]. Energy flow (EF) serves as a quantifiable metric for assessing mechanical energy transfer across body segments. Efficient energy flow patterns are closely linked to reduced injury risk and improved athletic performance [11]. Simon Augustus et al. [9] utilized energy flow analysis to investigate the energy transfer patterns between the support leg and the kicking leg during football instep kicking, providing empirical support for targeted training. Additionally, Gary L et al. [12] found that energy demand increased in individuals with lower back pain during STS transitions, potentially exacerbating their pain. However, whether changes in the BMI alter energy transfer strategies, leading to excessive energy demands in specific body segments, remains unclear.

Among various dynamic tasks, sit-to-stand (STS) transitions represent a fundamental daily activity performed over 50 times a day, serving as a reliable indicator of lower-limb functional status across diverse populations [13,14]. Given its functional significance, this study investigates the differences in limb coordination patterns and energy flow efficiency during the STS process among individuals with varying BMIs. This analysis aims to offer theoretical insights into the impact of BMI variations on movement mechanisms and inform targeted health interventions. This study hypothesizes that variations in BMI influence limb coordination patterns and energy transfer efficiency during STS transitions. Specifically, overweight individuals are expected to rely more on proximal lower-limb force generation, whereas underweight individuals may exhibit greater trunk compensation to accomplish the movement.

## 2. Materials and Methods

This study recruited forty participants, with the recruitment period starting on 28 April 2024 and ending on 15 June 2024. All participants signed a written informed consent form. Forty participants were categorized into three groups based on their BMI: the overweight group (overweight, OW), the normal weight group (normal weight, NW), and the underweight group (underweight, UW). All participants completed the sit-to-stand test. This study was approved by the Ethics Committee of Sports Science Experiments at Beijing Sport University (Approval Number: 2024120H).

### 2.1. Participants

A total of 40 participants (18–30 years) were recruited for the STS tests and categorized based on their BMI. Participants with a BMI ranging from 25.0 to 29.9 kg/m^2^ were assigned to the overweight group (OW), those with a BMI ranging from 18.5 to 24.9 kg/m^2^ were classified as the normal weight group (NW), and those with a BMI below 18.5 kg/m^2^ were placed in the underweight group (UW). The basic demographic information of the participants is presented in Table 1. The inclusion criteria were as follows: participants with no systematic or specialized sports training; those with sensory or neurological impairments; individuals with a history of patellar dislocation, lower-limb joint surgery, meniscus injury, or ligament damage; and those who had engaged in intense exercise or muscle fatigue within 72 h prior to testing.

There were no significant intergroup differences in age and gender distribution among the OW, NW, and UW groups (*p* > 0.05). However, significant differences were observed in height, weight, and BMI (*p* < 0.05). Specifically, in terms of height, the OW group was significantly taller than the UW group (*p* = 0.021), and the NW group was also taller than the UW group (*p* = 0.045). Regarding weight and BMI, the OW group exhibited a significantly higher weight and BMI compared to both the NW group (*p* < 0.001) and the UW group (*p* < 0.001). Additionally, the NW group had a higher weight and BMI than the UW group (*p* = 0.003, *p* = 0.002).

### 2.2. Data Collection

The experiment was conducted at the Rehabilitation and Sports Medicine Center of Beijing Sport University using an 8-camera infrared motion capture system (Qualisys, Sweden; sampling frequency: 200 Hz) and four 3D force plates (Kistler, Switzerland; sampling frequency: 1000 Hz). The participants were instructed to wear tight-fitting clothing and perform tests barefoot. Reflective markers were attached by trained personnel to anatomical landmarks, including the head, upper limbs, trunk (C7 spinous process, jugular notch, T10 spinous process, and xiphoid process), pelvis (anterior superior iliac spines, posterior superior iliac spines, and sacrum), and lower limbs (thigh markers, medial and lateral femoral condyles, tibial tuberosity, medial and lateral malleoli, heel, and distal ends of the 2nd and 5th metatarsals). Additionally, the height and weight of the participants were measured to calculate the BMI. Height was measured using a mechanical stadiometer (Seca, Germany, accuracy ±0.1 cm), and weight was measured using a weighing scale (Tanita, Japan, accuracy ±0.1 kg).

#### 2.2.1. Testing Procedure

Before testing, a height-adjustable armless and backless chair was placed on two force plates, with the chair height adjusted to 100% of the participant’s lower leg length. The participants were required to maintain the following initial posture: hands placed on the abdomen, trunk perpendicular to the ground, feet positioned on the force plates at hip-width apart with the toes pointing outward at a 15° angle, ankle dorsiflexion at 75°, and a sitting depth adjusted to half the length of the thigh, aligned with the front edge of the seat.

For the formal test, the participants sat stably for 5 s before standing up on verbal instruction from the examiner. They were instructed to stand at their natural and comfortable pace without moving their feet during the process, maintaining an upright posture for 15 s after standing. After each trial, the initial posture was reset to ensure standardized positioning. Five valid trials were collected for each participant.

#### 2.2.2. Phases of the STS Task

**Figure 1:**
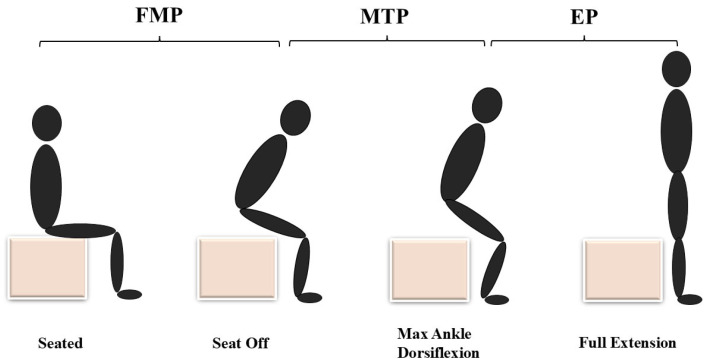
Schematic representation of the sit-to-stand (STS). FMP, the Flexion Momentum Phase; MTP, the Momentum Transfer Phase; EP, the Extension Phase.

The STS process was divided into three phases [15]: the Flexion Momentum Phase (FMP), the Momentum Transfer Phase (MTP), and the Extension Phase (EP), as illustrated in Figure 1. The definitions of each phase are as follows: Flexion Momentum Phase (FMP): Begins when the movement starts (indicated by a shoulder velocity exceeding 0.1 m/s) and ends just before the buttocks leave the seat (indicated by a ground reaction force under the chair dropping below 10 N). Momentum Transfer Phase (MTP): Starts when the buttocks leave the seat and ends at the point of maximum ankle dorsiflexion. During this phase, the momentum generated by the upper body in the FMP is transferred to the entire body, facilitating upward and forward motion. Extension Phase (EP): Begins after maximum ankle dorsiflexion and ends when the trunk is fully extended (indicated by a shoulder velocity dropping below 0.01 m/s).

### 2.3. Data Analysis

Motion Coordination Data Processing: The spatial coordinates of the reflective markers were identified using Qualisys Track Manager 2023. A fourth-order Butterworth low-pass filter with a cutoff frequency of 6 Hz was applied for noise reduction. The processed data were exported in .C3D format and were used to construct a human body model in Visual 3D 4.0 (C-Motion, Inc., Germantown, MD, USA). The trunk and pelvis were modeled as two independent rigid bodies, with their coordinate systems determined by the positions of the reflective markers. The calculations included angles, angular velocities, and linear velocities of the trunk and pelvis, and the proximal and distal joint forces. The lower-imb joint parameters included angles, torques, and joint forces for the hip, knee, and ankle, as well as angular and linear velocities of the thigh and shank.

The coupling angle was derived from the resultant angle formed by two consecutive data points on an angle–angle plot relative to the horizontal plane. The coupling angle was calculated using an improved vector coding technique [8,16]. For each frame (*i*) in the standardized sit-to-stand cycle, the coupling angle ($\gamma_{i}$) was computed using consecutive proximal segment angles ($\theta_{Di}$, $\theta_{D\left(i+1\right)}$) and the distal segment angle ($\theta_{Pi}$, $\theta_{P\left(i+1\right)}$) as follows:

(1) $$\gamma_{i}=Atan\left(\frac{\theta_{D\left(i+1\right)}-\theta_{Di}}{\theta_{P\left(i+1\right)}-\theta_{Pi}}\right).\frac{180}{\pi }\;\theta_{P\left(i+1\right)}-\theta_{Pi}>0$$

(2) $$\gamma_{i}=Atan\left(\frac{\theta_{D\left(i+1\right)}-\theta_{Di}}{\theta_{P\left(i+1\right)}-\theta_{Pi}}\right).\frac{180}{\pi }+180\;\theta_{P\left(i+1\right)}-\theta_{Pi}<0$$

The conditions for $\gamma_{i}$ are as follows:

(3) $$\gamma_{i}=\left\{\begin{array}{cccc} \gamma_{i}=90 & \theta_{P\left(i+1\right)}-\theta_{Pi}=0 & and & \theta_{D\left(i+1\right)}-\theta_{Di}>0\\ \gamma_{i}=-90 & \theta_{P\left(i+1\right)}-\theta_{Pi}=0 & and & \theta_{D\left(i+1\right)}-\theta_{Di}<0\\ \gamma_{i}=-180 & \theta_{P\left(i+1\right)}-\theta_{Pi}<0 & and & \theta_{D\left(i+1\right)}-\theta_{Di}=0\\ \gamma_{i}=Undefined & \theta_{P\left(i+1\right)}-\theta_{Pi}=0 & and & \theta_{D\left(i+1\right)}-\theta_{Di}=0 \end{array}\right.$$

After correction, the coupling angle$\;\gamma_{i}$ is constrained to lie between 0°and 360°:

(4) $$\gamma_{i}=\left\{\begin{array}{l} \gamma_{i}+360\;\;\;\;\gamma_{i}<0\\ \gamma_{i}\;\;\;\;\gamma_{i}\geq 0 \end{array}\right.$$

The formula for calculating the mean coupling angle length ${\overset{¯}{r}}_{i}$ is as follows:

(5) $${\overset{¯}{r}}_{i}=\sqrt{{\overset{¯}{x}}_{i}^{2}+{\overset{¯}{y}}_{i}^{2}}$$

The formula for calculating the Coupling Angle Variability $CAV_{i}\;$is as follows:

(6) $$CAV_{i}=\sqrt{2.(1-{\overset{¯}{r}}_{i})}.\frac{180}{\pi }.$$

Using MATLAB (MATLAB version 2020a; MathWorks; Natick, MA, USA), custom scripts were developed to calculate the coupling angles and mean variability for the hip-knee, knee-ankle, and hip-ankle joints of the lower limbs based on circular statistics. Coordination patterns were classified based on the coupling angles between body segments into in-phase, anti-phase, distal-phase, or proximal-phase coordination [17]. Details of the coordination pattern classifications are provided in Table 2. The frequency of each coordination pattern during each phase was calculated as a percentage, and the coordination variability was determined using circular statistics to represent the standard deviation of the coupling angles [7,16].

Energy Flow Data Processing: Power represents the energy flow rate between the segments per unit of time, indicating the speed of energy transfer. The total power flow within each body segment comprises active and passive mechanisms at the segment’s proximal and distal ends [18]. Passive power flow refers to the energy transfer through joint reaction forces and joint translation velocities. Active power flow refers to the energy transfer generated by muscle work. Positive segment power indicates the energy input into the segment, while negative segment power indicates the energy output from the segment [9,12].

Joint Power (*JP*): The rate of work done by joint forces on the segment, representing energy passively transferred into (positive power) or out of (negative power) adjacent segments. Where *Fj* is the joint force, and *vj* is the linear velocity at the joint center.

(7) JP=Fj⋅νj

Muscle Power (*MP*): The scalar product of joint torque and the angular velocity of the respective segment, where *Mj* is the joint torque and *ωs* is the segment angular velocity.

(8) MP=Mj·ωs

Segment Power (*SP*): The net energy input or output for a segment, calculated as the sum of the joint power and muscle power at the proximal and distal ends of the segment. Where *JPd* and *JPp* are the distal and proximal joint powers, and *MPd* and *MPp* are the distal and proximal muscle powers.

(9) SP=JPd+JPp+MPd+MPp

### 2.4. Statistical Analysis

In this study, data for coordination patterns, coordination frequency, JP, MP, and SP were tested for normality using the Shapiro–Wilk test. Data with normal distributions were analyzed using one-way ANOVA. Non-normally distributed data were analyzed using the Kruskal–Wallis test. Post hoc pairwise comparisons were conducted using the Bonferroni test. A significance level of *p* < 0.05 was applied. Statistical analyses were performed using SPSS 22.0 (IBM Corporation, Somers, NY, USA).

## 3. Results

### 3.1. Coordination Characteristics Analysis in STS

**Figure 2:**
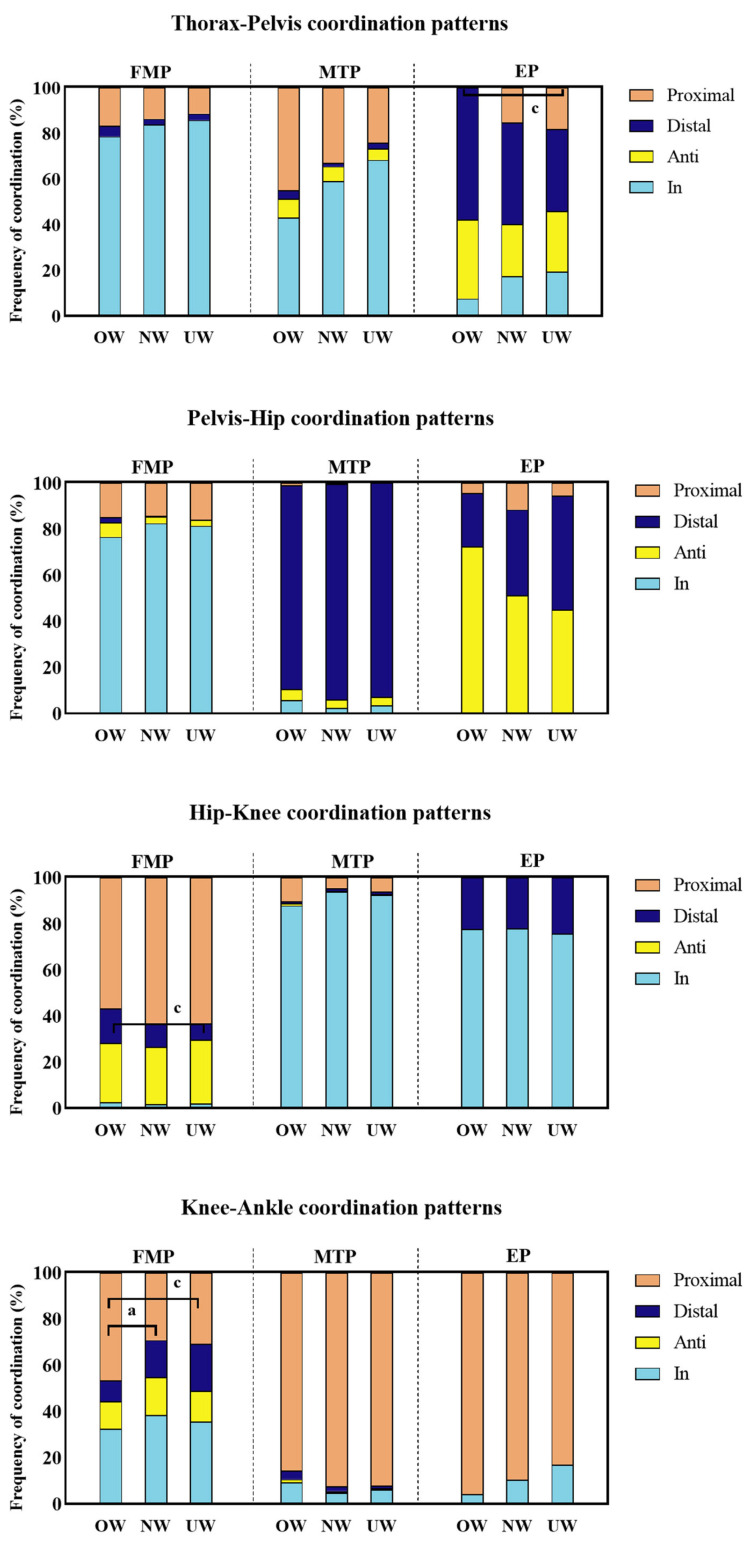

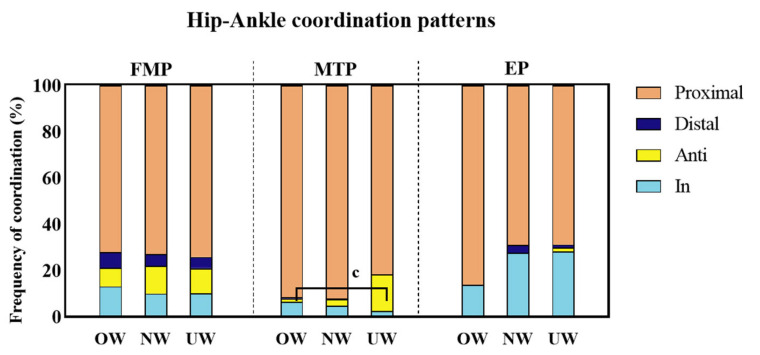
Sagittal plane coordination patterns. FMP, the Flexion Momentum Phase; MTP, the Momentum Transfer Phase; EP, the Extension Phase; OW, the overweight group; NW, the normal weight group; UW, the underweight group. Differences were compared by one-way ANOVA. Significant differences are indicated by “a” (NW vs. OW) and “c” (UW vs. OW) (*p* < 0.05).

**Figure 3:**
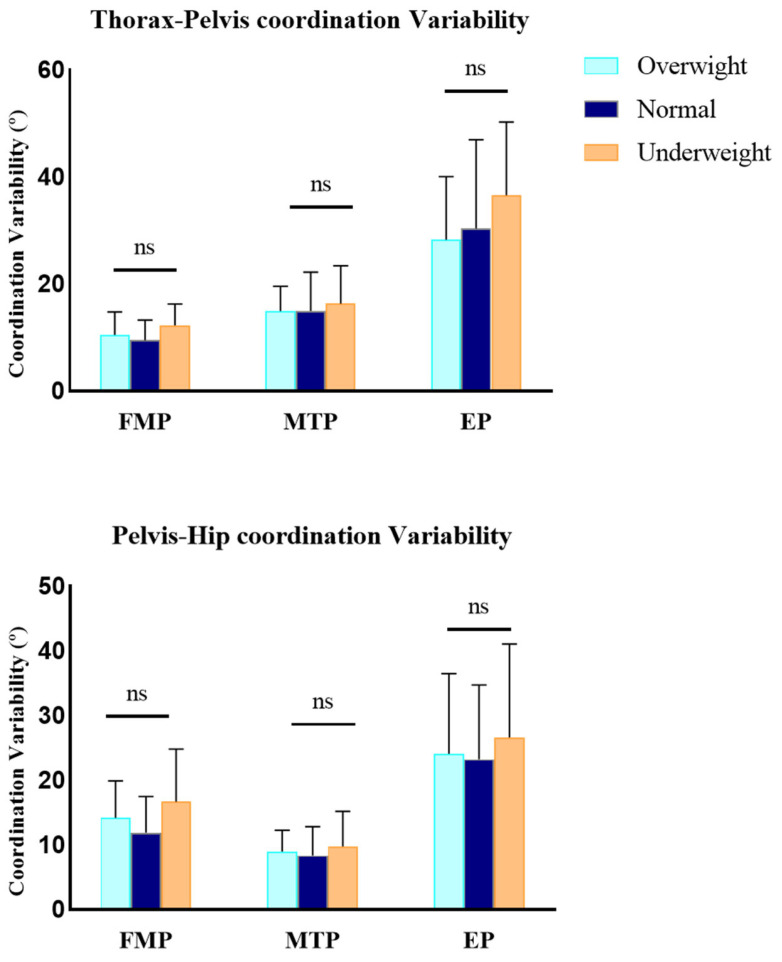

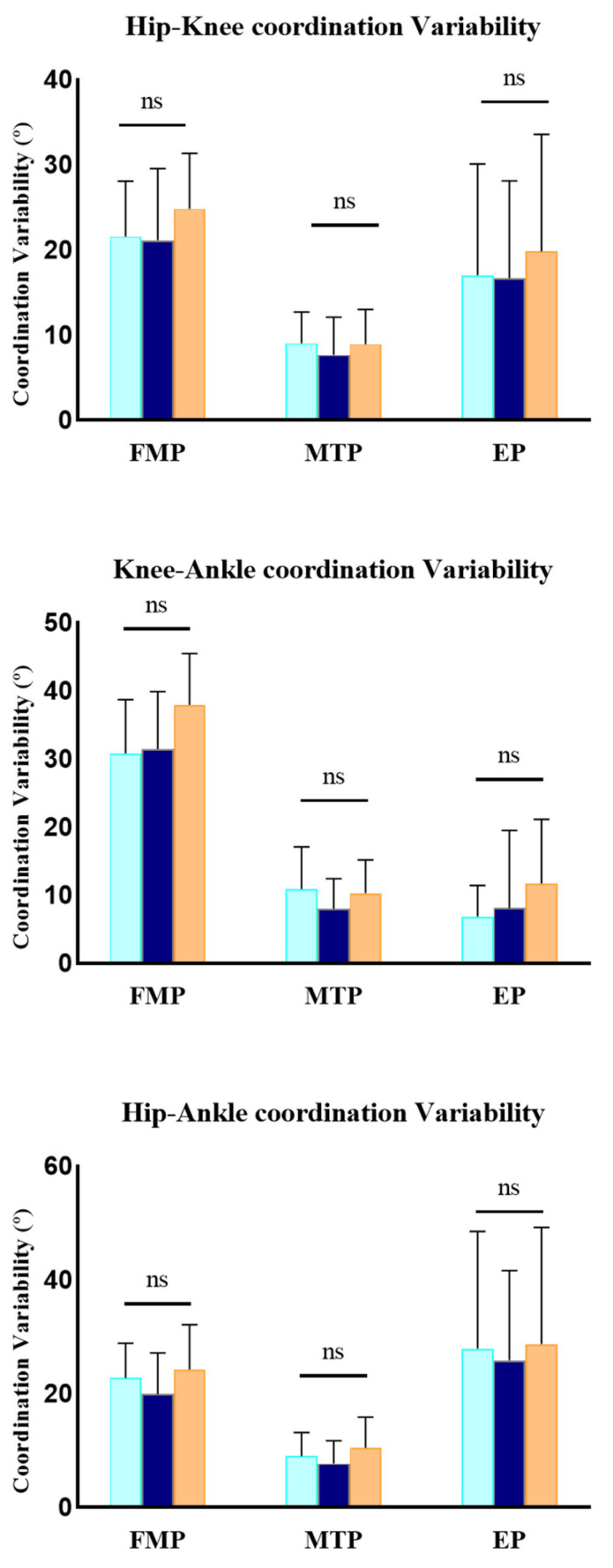
Sagittal plane coordination variability. Differences were compared by one-way ANOVA. “ns” denotes non-significant differences. FMP, the Flexion Momentum Phase; MTP, the Momentum Transfer Phase; EP, the Extension Phase.

The frequency of the sagittal plane coordination patterns exhibited significant intergroup differences (*p* < 0.05), whereas the coordination variability showed no statistical significance (*p* > 0.05) (Figure 2 and Figure 3). In the FMP phase, the overweight group showed a higher frequency of distal coordination patterns in the hip-knee coupling compared to the underweight group (F_(2,35)_ = 6.033, *p*=0.005). For the knee-ankle coupling, the overweight group demonstrated a higher frequency of proximal coordination patterns compared to the normal weight and underweight groups (F_(2,35)_ = 5.509, OW vs. NW: *p* = 0.008; OW vs. UW: *p* = 0.044). In the MTP phase, the overweight group exhibited a lower frequency of anti-phase coordination patterns in the hip-ankle coupling compared to the underweight group (F_(2,35)_= 5.051, *p* = 0.043). In the EP phase, the overweight group demonstrated a lower frequency of proximal coordination patterns in the trunk-pelvis coupling compared to the underweight group (F_(2,35)_= 3.272, *p* = 0.01).

### 3.2. Energy Flow in STS

**Figure 4:**
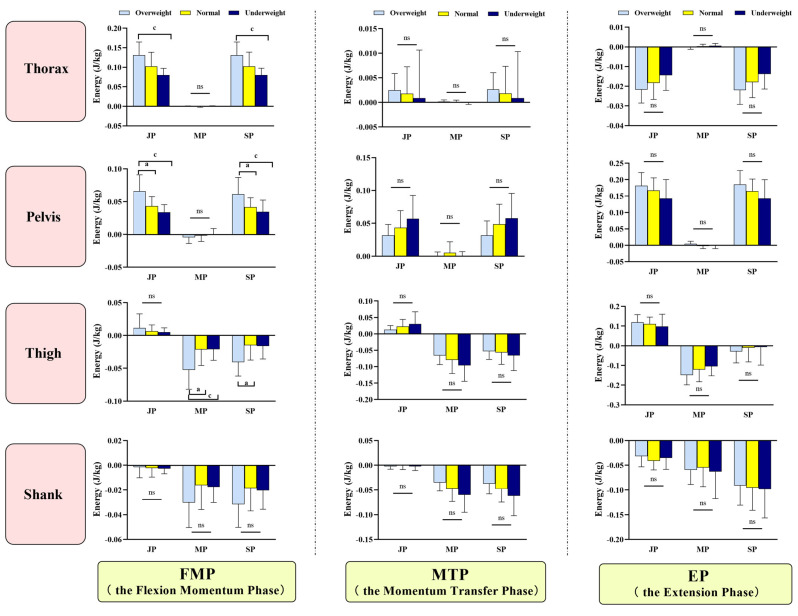
Schematic representation of energy flow patterns in sit-to-stand (STS). JP, joint power; MP, muscle power; SP, segment power. Differences were compared by one-way ANOVA. Significant differences are indicated by “a” (NW vs. OW) and “c” (UW vs. OW) (*p* < 0.05), while “ns” denotes non-significance.

The energy flow across body segments varied significantly among individuals with different body weights (Figure 4). In the FMP phase trunk segment, the overweight group exhibited a higher JP (*F*_(2,33)_ = 6.238, *p* = 0.004) and SP compared to the underweight group (*F*_(2,33)_ = 6.134, *p*=0.004). Pelvis segment: The overweight group showed a higher JP (*F*_(2,33)_ = 8.836, *OW* vs. *NW: p* = 0.009; *OW* vs. *UW: p* = 0.001) and SP (F_(2,33)_ = 5.360, OW vs. NW: *p* = 0.044; OW vs. UW: *p* = 0.01) compared to both the normal weight and underweight groups. Thigh segment: The overweight group demonstrated a higher MP compared to the normal weight and underweight groups (F_(2,33)_ = 5.630, OW vs. NW: *p* = 0.011; OW vs. UW: *p* = 0.019) and a higher SP compared to the normal weight group (*F*_(2,33)_ = 4.711, *p* = 0.024).

## 4. Discussion

STS is a highly frequent daily activity that imposes significant load demands on the lower limbs. However, purely kinematic analysis limits our understanding of how body segments coordinate posture and distribute loads. Therefore, this study analyzed intersegmental coordination strategies and energy flow patterns in overweight, normal weight, and underweight populations during STS using a combination of motion coordination and energy transfer approaches.

This study found differences in the sagittal plane coordination patterns among overweight, underweight, and normal weight populations during STS. In the FMP phase, the overweight group exhibited a higher frequency of distal coordination patterns in the hip-knee coupling compared to the underweight group and borderline significantly higher than the normal weight group (*p* = 0.052). For the knee-ankle coupling, the overweight group demonstrated a higher frequency of proximal coordination patterns than the normal weight and underweight groups, indicating that in the initiation phase of STS, overweight individuals predominantly rely on knee joint drive. Previous studies have shown that individuals with a higher BMI exhibit greater knee flexion torque compared to hip torque during STS [14]. This study also found that the distal coordination pattern in the hip-knee coupling and the proximal coordination pattern in the knee-ankle coupling were both characterized by knee joint drive dominance during movement initiation, with relatively insufficient hip joint strength. In terms of energy flow, the thigh segment in the overweight group showed significantly higher MP output compared to the normal weight and underweight groups, indicating that the thigh muscles in overweight individuals need to produce higher power output to compensate for the lack of hip joint drive, ensuring successful movement completion. Although this pattern meets the immediate needs of movement initiation, it may increase the mechanical load on the knee joint over time, potentially raising the risk of knee injuries. Furthermore, in energy flow theory, JP represents passive forces within a segment, referring to the interaction forces between joints, while MP represents active forces within a segment, referring to neuromuscular forces [9,12,19]. During the initiation phase of STS, the joint power and segmental net power in the trunk and pelvis segments were higher in the overweight group compared to the underweight group, indicating that overweight individuals require more energy to maintain balance and stability during trunk movement execution. This is likely due to the greater body weight in the overweight group, necessitating higher energy output to overcome inertia and gravitational forces during the movement process, thereby ensuring the smooth execution of the action [20].

In the MTP phase, the frequency of anti-phase coordination patterns in the hip-ankle coupling was higher in the underweight group compared to the overweight group and borderline significantly higher than that in the normal BMI group (*p* = 0.052). This suggests that underweight individuals frequently adjust hip and ankle movements to maintain balance or complete the movement task. Studies have shown that the human body primarily relies on coordinated movements of the ankle and hip joints to maintain balance during standing, and under significant external perturbations, the body tends to adjust hip joint movements to maintain the center of mass and ensure stability [21,22]. In the energy flow analysis, the underweight group showed significantly higher lower-limb muscle power output (Figure 4). This indicates that, compared to normal and overweight groups, the underweight group relies more on increasing lower-limb muscle power output to adjust momentum transfer and control the center of mass during the movement process, maintaining efficient and stable movement.

In the EP phase, the frequency of proximal coordination patterns in the trunk-pelvis coupling was lower in the overweight group than in the underweight group, and the frequency of proximal coordination patterns in the overweight group was borderline significantly lower than in the normal group (*p* = 0.062). In terms of trunk-pelvis coordination, the normal and underweight groups exhibited similar patterns. Both groups needed more coordination modes in the EP phase to maintain body stability, especially the close coordination between the trunk and pelvis played a crucial role in the movement process. Due to their lighter or normal body weight, the low body weight and normal weight groups were able to effectively utilize close coordination between the trunk and pelvis to improve stability during movement. Studies have shown that BMI significantly impacts balance function, with higher BMI groups showing poorer dynamic balance, suggesting that individuals with a higher BMI may adopt different coordination strategies in posture control to cope with the challenges posed by increased body weight [23,24]. In addition, the underweight group exhibited more proximal coordination patterns during this phase, indicating their ability to flexibly adjust trunk-pelvis coordination without heavily relying on other lower-limb coordination patterns to compensate for lack of body stability [25]. Unlike the underweight and normal groups, the overweight group displayed fewer proximal coordination patterns in the trunk-pelvis coordination during the EP phase. Due to their higher body weight, the overweight group likely relies more on a pelvic-driven movement pattern, reducing the tight coordination between the trunk and pelvis. Additionally, the pelvic-hip coordination pattern in the overweight group predominantly exhibited anti-phase coordination. This anti-phase coordination optimizes center-of-mass control and stability through the opposing movements between the pelvis and hip joint, reducing the load on the trunk and stabilizing the center of mass during the movement, thus minimizing the effects of inertia [26].

This study systematically analyzes coordination patterns and energy flow characteristics during the STS process, revealing differences in motor control among individuals with different BMI classifications. It provides important theoretical support for understanding the impact of body weight on sit-to-stand movement patterns. The findings not only have academic value but also hold strong practical significance, offering scientific insights for physical trainers and rehabilitation professionals to develop personalized training and rehabilitation strategies for individuals with varying BMI levels. Furthermore, by comparing coordination patterns and energy flow characteristics across BMI groups, this study deepens our understanding of movement efficiency and postural control, providing valuable support for optimizing performance, enhancing movement stability, and preventing injuries.

The limitations of this study are as follows: First, while comparisons were made between different BMI groups, the lack of an obese sample may limit the generalizability of the results. Future studies should include obese individuals for comparison. Second, the study did not incorporate electromyography (EMG) data, which would provide deeper insights into muscle activity differences across BMI groups during the sit-to-stand (STS) process. Future research can use EMG data to reveal differences in movement patterns, which would help design personalized rehabilitation programs. Third, this study focused solely on young adults and did not consider how age may affect sit-stand posture patterns. Future studies should include elderly individuals for comparison to understand how sit-stand patterns evolve with age. Lastly, while this study included different BMI groups, the sample size was relatively small, and the sample diversity was limited. Future research should expand the sample size and include individuals from diverse ethnic, gender, and socioeconomic backgrounds to enhance the external validity of the findings.

## 5. Conclusions

Changes in BMI affect movement coordination and energy transfer strategies during STS. OW individuals compensate for insufficient hip drive by relying on trunk and pelvic power, which may increase knee and trunk load over time. In contrast, UW individuals exhibit greater lower-limb flexibility and rely on trunk-pelvis coordination to compensate for stability deficits. Future research should develop targeted exercise interventions to optimize movement patterns and reduce injury risk across BMI groups.

## Figures and Tables

**Table 1:** Characteristics of the participants (Mean ± SD).

	NW	OW	UW	Statistical Results
Gender (F/M)	9/11	2/8	6/4	χ^2^ = 6.735, *p* = 0.151
Age (y)	23 ± 2.13	24.56 ± 2.55	22.5 ± 2.12	F = 1.214, *p* = 0.311
Height (cm)	171.16 ± 8.54	176.07 ± 6.99	167.13 ± 7.52	F = 3.785, *p* = 0.034
Weight (kg)	62.26 ± 8.21	82.96 ± 7.7	50.54 ± 4.71	F = 27.906, *p* < 0.001
Body mass index (kg/m^2^)	21.17 ± 1.21	26.73 ± 1.44	18.06 ± 0.32	F = 30.472, *p* < 0.001

Abbreviations: NW, normal weight; OW, overweight; UW, underweight.

**Table 2:** Classification of coordination patterns.

Coordination Patterns	45° Bins
In-Phase	22.5° ≤ γ < 67.5°, 202.5° ≤ γ < 247.5°
Anti-Phase	112.5° ≤ γ < 157.5°, 292.5° ≤ γ < 337.5°
Proximal-Phase	337.5° ≤ γ < 22.5°, 157.5° ≤ γ < 202.5°
Distal-Phase	67.5° ≤ γ < 112.5°, 247.5° ≤ γ < 292.5°

Abbreviations: γ, coupling angle.

## Data Availability

The raw data supporting the conclusions of this article are available from the first author upon reasonable request.
